# Berberine Ameliorates Hepatic Steatosis and Suppresses Liver and Adipose Tissue Inflammation in Mice with Diet-induced Obesity

**DOI:** 10.1038/srep22612

**Published:** 2016-03-03

**Authors:** Ting Guo, Shih-Lung Woo, Xin Guo, Honggui Li, Juan Zheng, Rachel Botchlett, Mengyang Liu, Ya Pei, Hang Xu, Yuli Cai, Tianshu Zeng, Lulu Chen, Xiaodong Li, Qifu Li, Xiaoqiu Xiao, Yuqing Huo, Chaodong Wu

**Affiliations:** 1 Department of Nutrition and Food Science, Texas A&M University, College Station, TX 77843, USA; 2 Department of Endocrinology, Union Hospital, Tongji Medical College, Huazhong University of Science and Technology, Wuhan, Hubei 430022, China; 3 Institute of Hepatology, Hospital of Chinese Medicine of Hubei, Hubei University of Chinese Medicine, Hubei 430060, China; 4 Department of Endocrinology, the First Affiliated Hospital of Chongqing Medical University, Chongqing 400016, China; 5 The Laboratory of Lipid & Glucose Metabolism, the First Affiliated Hospital of Chongqing Medical University, Chongqing 400016, China; 6 Vascular Biology Center, Department of Cellular Biology and Anatomy, Medical College of Georgia, Georgia Regents University, Augusta, GA 30912, USA; 7 Drug Discovery Center, Key Laboratory of Chemical Genomics, Peking University Shenzhen Graduate School, Shenzhen 518055, China

## Abstract

Increasing evidence demonstrates that berberine (BBR) is beneficial for obesity-associated non-alcoholic fatty liver disease (NAFLD). However, it remains to be elucidated how BBR improves aspects of NAFLD. Here we revealed an AMP-activated protein kinase (AMPK)-independent mechanism for BBR to suppress obesity-associated inflammation and improve hepatic steatosis. In C57BL/6J mice fed a high-fat diet (HFD), treatment with BBR decreased inflammation in both the liver and adipose tissue as indicated by reduction of the phosphorylation state of JNK1 and the mRNA levels of proinflammatory cytokines. BBR treatment also decreased hepatic steatosis, as well as the expression of acetyl-CoA carboxylase and fatty acid synthase. Interestingly, treatment with BBR did not significantly alter the phosphorylation state of AMPK in both the liver and adipose tissue of HFD-fed mice. Consistently, BBR treatment significantly decreased the phosphorylation state of JNK1 in both hepatoma H4IIE cells and mouse primary hepatocytes in both dose-dependent and time-dependent manners, which was independent of AMPK phosphorylation. BBR treatment also caused a decrease in palmitate-induced fat deposition in primary mouse hepatocytes. Taken together, these results suggest that BBR actions on improving aspects of NAFLD are largely attributable to BBR suppression of inflammation, which is independent of AMPK.

Non-alcoholic fatty liver disease (NAFLD) encompasses the whole spectrum of liver diseases including hepatic steatosis, non-alcoholic steatohepatitis (NASH), and cirrhosis that are not associated with significant alcohol consumption^1^. While simple steatosis is largely considered benign, NAFLD could progress to its advanced stage, NASH, if the liver displays overt necroinflammation, and could eventually progress to cirrhosis, liver failure and liver cancer^2^,^3^,^4^. Many epidemiological studies have demonstrated that the incidence of NAFLD is markedly increased in obese populations^1^,^5^,^6^. Consistently, in rodent models of diet-induced obesity (DIO), hepatic steatosis and inflammation are characteristics that are commonly associated with systemic insulin resistance and glucose metabolic dysregulation. Indeed, hepatic steatosis is sufficient to trigger inflammatory responses^7^,^8^ and to induce liver insulin resistance by activating protein kinase Cε^9^. Furthermore, hepatic steatosis is a major contributor of dyslipidemia that works with or without insulin resistance to significantly increase the incidence of atherogenic cardiovascular diseases^10^. Because of this, NAFLD is considered a critical component of metabolic syndrome.

The exact mechanisms underlying the pathogenesis of NAFLD remain to be explored. Because of the close relationship between obesity and NAFLD, obesity-associated inflammation and insulin resistance are accepted as key factors that initiate or exacerbate NAFLD. For instance, both inflammation and insulin resistance can cause hepatic steatosis through increasing liver expression of genes for lipogenic enzymes such as acetyl-CoA carboxylase 1 (ACC1) and fatty acid synthase (FAS) and decreasing liver expression of genes for fatty acid oxidation including carnitine palmitoyltransferase 1a (CPT1a)^11^,^12^,^13^. In terms of generating inflammatory mediators, adipose tissue has been implicated as an essential location where a number of proinflammatory cytokines are released and, in turn, bring about the secondary effects on the liver to serve as a “second hit” to exacerbate NAFLD during obesity^14^. Interestingly, adipose tissue also releases palmitoleate, which, at increased levels, stimulates hepatic steatosis, but suppresses liver inflammatory responses^15^,^16^. Clearly, the inflammatory status, at both the liver and systemic levels, is of particular importance to the development and progression of NAFLD, and this inflammation can occur independent of fat deposition^15^,^16^.

Berberine (BBR), a natural compound found in numerous herb plants including *Coptis Chinensis*, has received much attention as many animal studies and clinical trials have reported anti-hyperglycemia and anti-dyslipidemia effects of BBR^17^,^18^,^19^,^20^. Of interest, BBR also displays a potent effect on reducing hepatic steatosis as supported by the results from both animal and clinical investigations^19^,^20^,^21^. This anti-hepatic steatosis effect of BBR is attributable to, at least in part, the actions of BBR on reducing the methylation of the microsomal triglyceride transfer protein (MTTP) promoter^19^ and on decreasing the DNA demethylation and histone acetylation in regulation of L-pyruvate kinase^22^. Because of the essential role played by AMP-activated protein kinase (AMPK) in fat metabolism^23^, a number of studies have also demonstrated AMPK as a critical mediator responsible for the anti-hepatic steatosis effect of BBR^21^. However, the extent to which the anti-inflammatory effect of BBR contributes to its anti-NAFLD effect remains largely unknown; although BBR is well recognized as a classic anti-inflammatory natural compound and is shown to inhibit hepatocyte inflammatory responses^24^. Considering the importance of inflammation in the development and progression of NAFLD, the present study examined the anti-inflammatory effect of BBR in the context of improving NAFLD. Our novel findings suggested an AMPK-independent mechanism for BBR actions on improving obesity-associated hepatic steatosis and inflammation.

## Results

### Berberine ameliorates obesity-associated systemic insulin resistance and glucose intolerance

**Figure 1:**
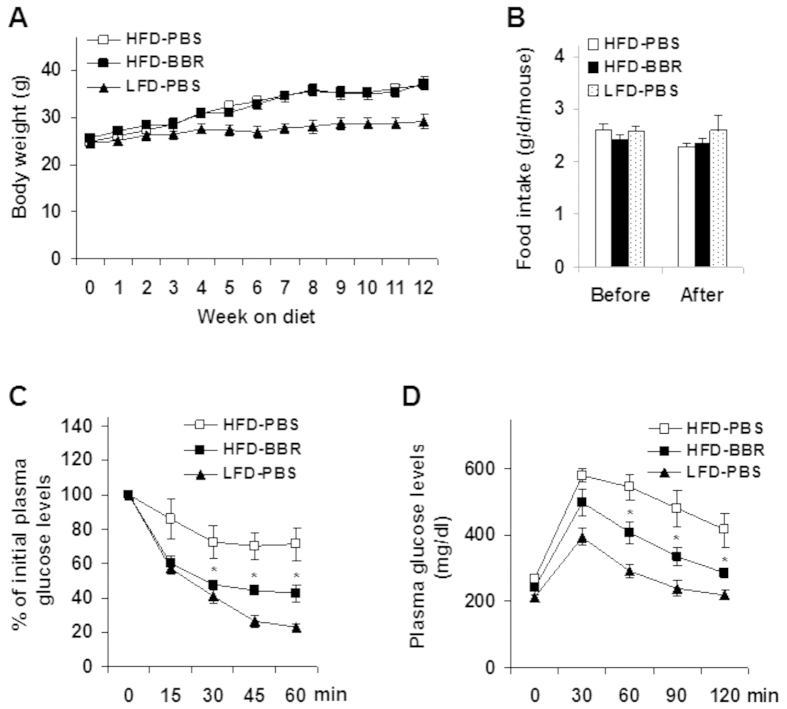
Berberine treatment ameliorates HFD-induced insulin resistance and glucose intolerance. Male C57BL/6J mice, at 5–6 weeks of age, were fed a high-fat diet (HFD) for 12 weeks and treated with berberine (BBR, 100 mg/kg/d, in phosphate-buffered saline (PBS)) or PBS for the last 4 weeks of HFD feeding. Age- and gender-matched C57BL/6J mice were fed a low-fat diet (LFD) for 12 weeks and treated with PBS for the last 4 weeks of LFD feeding. Data are means ± SE, n = 8–10. (**A**) Body weight was monitored weekly during the feeding/treatment period. (**B**) Food intake was calculated based on food consumption per day per mouse. (**C**) Insulin tolerance tests (ITT). (**D**) Glucose tolerance tests (GTT). For C and D, mice were fasted for 4 hr and received an intraperitoneal injection of insulin (1 U/kg body weight) (**C**) or glucose (2 g/kg body weight) (**D**). *P < 0.05 HFD-BBR vs. HFD-PBS for the same time point (**C,D**).

Upon feeding of a high-fat diet (HFD) for 4 weeks, C57BL/6J mice started to demonstrate a significant increase in body weight (diet-induced obesity, DIO) compared with age- and gender-matched mice on a low-fat diet (LFD) (Fig. 1A). At the end of 12 week feeding/treatment period, HFD-fed and phosphate-buffered saline (PBS)-treated (HFD-PBS) mice gained much more body weight than LFD-fed and PBS-treated (LFD-PBS) mice (*P* < 0.001, HFD-PBS vs. LFD-PBS). Within HFD-fed mice, treatment with BBR did not alter body weight and food intake compared with HFD-PBS control mice (Fig. 1A,B). However, HFD-fed and BBR-treated (HFD-BBR) mice displayed a significant decrease in the severity of obesity-associated systemic insulin resistance and glucose intolerance (Fig. 1C,D) compared with HFD-PBS mice. These results suggest that BBR improves systemic insulin sensitivity and glucose homeostasis in the absence of altering body weight.

### Berberine decreases obesity-associated liver inflammation

**Figure 2:**
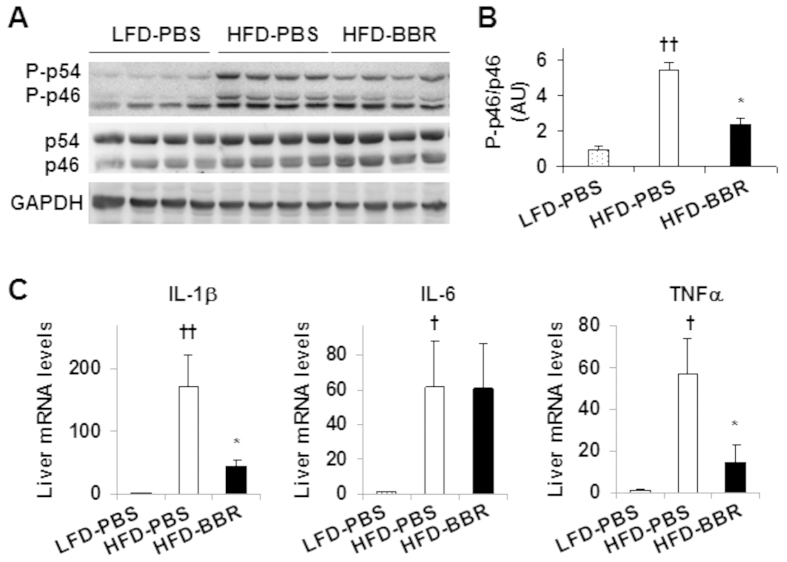
Berberine treatment decreases HFD-induced liver inflammatory responses. Mouse experiments were performed as descried in Fig. 1. (**A,B**) Liver inflammatory signaling. Liver extracts were subjected to Western blot analyses (**A**). Ratios of phosphorylated JNK1 to total JNK1 (P-p46/p46) were quantified using densitometry and normalized to GAPDH (**B**). AU, arbitrary unit. (**C**) Liver mRNA levels of pro-inflammatory cytokines were analyzed using quantitative real-time PCR. For bar graphs, data are means ± SE (n = 4–6). *P < 0.05, HFD-BBR vs. HFD-PBS; ^†^*P* < 0.05 and ^††^*P* < 0.01 HFD-PBS vs. LFD-PBS.

Since inflammation is a critical factor that drives the progression of simple steatosis to NASH^4^,^5^, we examined the inflammatory signaling through JNK and quantified the mRNA levels of proinflammatory cytokines to assess BBR action on the liver inflammatory status. Compared with that in livers of LFD-PBS mice, the phosphorylation state of JNK1 (p46) in livers of HFD-PBS mice showed a 5.5-fold increase (Fig. 2A,B). Upon treatment with BBR, the phosphorylation state of liver p46 was significantly decreased compared with that of HFD-PBS mice (56% decrease; Fig. 2A,B). Next, we examined the mRNA levels of proinflammatory cytokines including IL-1β, IL-6, and TNFα, which all were significantly increased in livers of HFD-PBS mice when compared with their respective levels in livers of LFD-PBS mice. Upon treatment with BBR, liver mRNA levels of IL-1β and TNFα were significantly decreased while liver mRNA levels of IL-6 were not significantly altered (Fig. 2C). These results suggest that treatment with BBR ameliorates obesity-associated liver inflammation.

### Berberine ameliorates obesity-associated hepatic steatosis without altering AMPK phosphorylation

**Figure 3:**
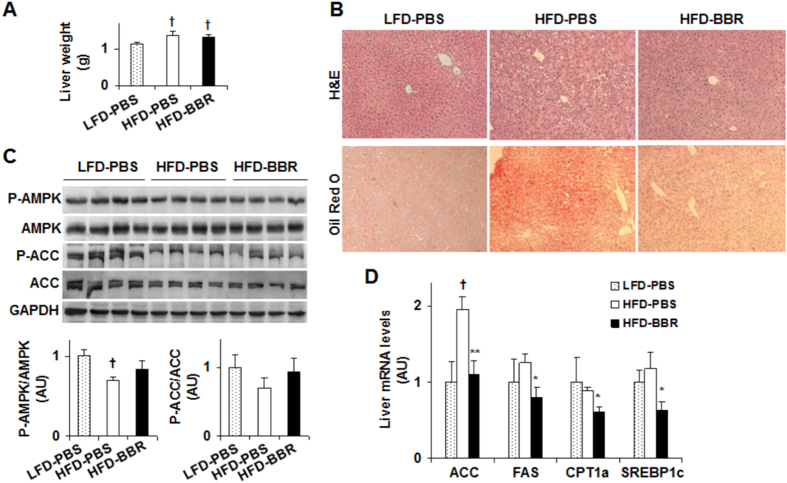
Berberine treatment ameliorates fat deposition in the liver and decrease hepatic expression of lipogenic genes. Mouse experiments were performed as descried in Fig. 1. (**A**) Liver weight. (**B**) Liver sections for H&E staining (top panels) and Oil Red O staining (bottom panels). (**C**) Liver AMPK signaling. Liver extracts were subjected to Western blot analyses. Ratios of phosphorylated AMPK to total AMPK (P-AMPK/AMPK) and phosphorylated ACC to total ACC (P-ACC/ACC) were quantified using densitometry and normalized to GAPDH (bar graphs). AU, arbitrary unit. (**D**) Liver mRNA levels of genes related to fat metabolism were analyzed using quantitative real-time PCR. For all bar graphs, data are means ± SE (n = 4–7). *P < 0.05 and **P < 0.01 HFD-BBR vs. HFD-PBS; ^†^*P* < 0.05 HFD-PBS or HFD-BBR vs. LFD-PBS.

Hepatic steatosis is a characteristic of NAFLD, whose incidence is markedly increased by obesity. We measured liver weight and did not observe significant differences between HFD-BBR mice and HFD-PBS mice (Fig. 3A); although liver weight of HFD-BBR mice or HFD-PBS mice was significantly greater than that of LFD-PBS mice. Compared with LFD-PBS mice, HFD-PBS mice displayed a marked increase in liver fat deposition, validating obesity-associated hepatic steatosis (Fig. 3B). However, the severity of obesity-associated hepatic steatosis was significantly decreased in HFD-BBR mice compared with that in HFD-PBS mice as indicated by results of H&E staining and Oil Red O staining. Since AMPK plays a critical role in the pathogenesis of hepatic steatosis^23^,^25^,^26^, we examined AMPK signaling. Compared with that in LFD-PBS mice, the phosphorylation state of liver AMPK in HFD-PBS mice was significantly decreased. This decrease, however, was not recovered by treatment with BBR (Fig. 3C); although BBR brought about a significant decrease in the severity of obesity-associated hepatic steatosis. Similar trends were also observed for the phosphorylation state of ACC. In contrast, treatment with BBR brought about a significant decrease in the mRNA levels of genes related to lipogenesis such as ACC, FAS, and sterol regulatory element-binding protein 1c (SREBP1c) although BBR decreased the mRNA levels of CPT1a, a rate-determining enzyme that favors fatty acid oxidation (Fig. 3D). These results suggest that BBR amelioration of obesity-associated hepatic steatosis involves in a decrease in liver lipogenesis, which is AMPK-independent.

### Berberine improves hepatocyte inflammatory and metabolic responses

**Figure 4:**
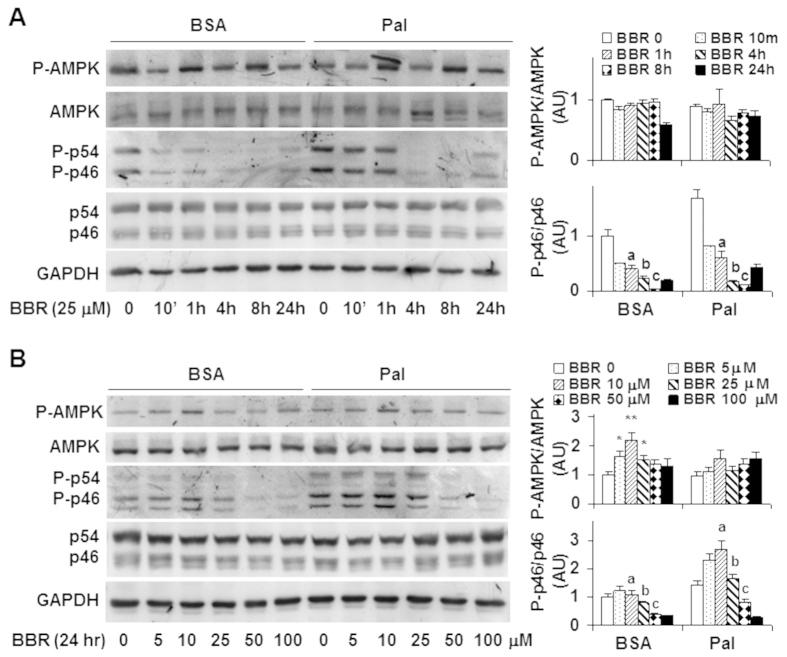
Berberine treatment decreases JNK1 phosphorylation while showing limited effects on AMPK phosphorylation in H4IIE cells. (**A**) Time-course study. H4IIE cells were pre-treated with palmitate (250 μM, conjugated with bovine serum albumin (BSA)) or BSA for 2 hr and then supplemented with berberine (BBR, 25 μM in PBS) or PBS for an additional 10 min, 1 hr, 4 hr, 8 hr, and/or 24 hr. (**B**) Dose-response study. H4IIE cells were pre-treated with palmitate (250 μM) or BSA for 2 hr and then supplemented with BBR or PBS for an additional 24 hr. For (**A**,**B**), cell extracts were subjected to Western blot analyses. Left panels, representative blots. For all bar graphs, ratios of phosphorylated AMPK to total AMPK (P-AMPK/AMPK) and phosphorylated JNK1 to total JNK1 (P-p46/p46) were quantified using densitometry and normalized to GAPDH. AU, arbitrary unit. Data are means ± SE (n = 4). *P < 0.05 and **P < 0.01 vs. BBR 0 (in **B**); Labeled means without a common letter under the same condition (BSA or Pal), *P* < 0.05 (in **A,B**).

To examine the direct effects of BBR on hepatocyte inflammatory and metabolic responses, we performed dose-response and time-course studies using H4IIE cells, a rat hepatoma cell line^26^. In the time-course study, H4IIE cells were treated with BBR at a dose of 25 μM for various time periods. Under basal (bovine serum albumin (BSA)-treated) conditions, BBR treatment significantly decreased the phosphorylation state of p46 at a time point as early as 10 min. Furthermore, the effect of BBR on decreasing the phosphorylation state of p46 displayed a time-dependent manner between 1 hr- and 8 hr-post BBR treatment (Fig. 4A). Under palmitate-stimulated conditions where the phosphorylation state of p46 was increased, treatment with BBR was still able to decrease palmitate-stimulated p46 phosphorylation, but required a relatively longer time period, i.e., at least 4 hr (Fig. 4A). Consistently, the effect of BBR on decreasing the phosphorylation state of p46 also revealed a time-dependent manner between 1 hr- and 8 hr-post BBR treatment under palmitate-stimulated conditions. In contrast, under both basal and palmitate-stimulated conditions, the phosphorylation state of hepatocyte AMPK did not differ significantly during the time period when BBR decreased the phosphorylation state of liver p46 in a time-dependent manner. In the dose-response study, treatment of H4IIE cells with BBR at a dose of 10 μM, 25 μM, or 50 μM for 24 hr decreased the phosphorylation state of p46 under basal conditions (Fig. 4B). Under palmitate-stimulated conditions, treatment of H4IIE cells with BBR was also able to decrease the phosphorylation state of JNK1 at a concentration of 50 μM or 100 μM (Fig. 4B). In particular, the effect of BBR on decreasing the phosphorylation state of p46 revealed a dose-dependent manner at the dose range between 10 μM and 50 μM, the conditions where BBR treatment did not significantly increase the phosphorylation state of hepatocyte AMPK. Therefore, these results suggest that the anti-inflammatory effect of BBR in H4IIE cells does not involve increased AMPK phosphorylation.

**Figure 5:**
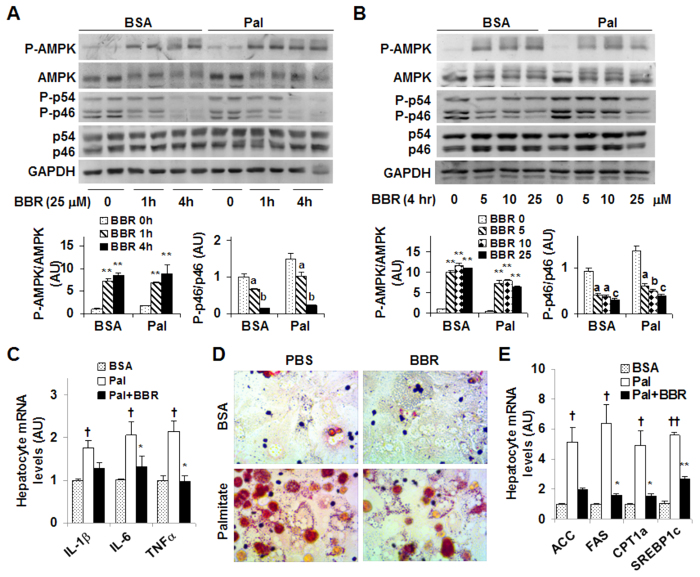
Berberine treatment suppresses the proinflammatory responses and ameliorates fat deposition in mouse primary hepatocytes while differentially altering AMPK. (**A**) Time-course study. Primary mouse hepatocytes were pre-treated with palmitate (250 μM, conjugated with bovine serum albumin (BSA)) or BSA for 2 hr and then supplemented with berberine (BBR, 25 μM in PBS) or PBS for an additional 1 hr and/or 4 hr. (**B**) Dose-response study. Primary mouse hepatocytes were pre-treated with palmitate (250 μM) or BSA for 2 hr and then supplemented with BBR at a dose of 5, 10, or 25 μM or PBS for an additional 4 hr. For (**A**,**B**), top panels, representative blots. Bar graphs, ratios of phosphorylated AMPK to total AMPK (P-AMPK/AMPK) and phosphorylated JNK1 to total JNK1 (P-p46/p46) were quantified using densitometry and normalized to GAPDH. AU, arbitrary unit. Data are means ± SE (n = 4). **P < 0.01 vs. BBR 0 (in **A**,**B**); Labeled means without a common letter under the same condition (BSA or Pal), *P* < 0.05. (**C**) Hepatocyte proinflammatory cytokine expression. (**D**) Hepatocyte fat deposition. (**E**) Hepatocyte mRNA levels of genes related to fat metabolism. For (**C–E**), primary mouse hepatocytes were as described in A with or without palmitate for 2 hr in the presence or absence of BBR for an additional 4 hr. For (**C**,**E**), the mRNA levels of pro-inflammatory cytokines and metabolic genes were analyzed using quantitative real-time PCR. For bar graphs in (**C**,**E**), data are means ± SE (n = 4–6). *P < 0.05 and **P < 0.01 Pal+BBR vs. Pal; ^†^*P *< 0.05 and ^††^*P* < 0.01 Pal vs. BSA.

Next, we used primary mouse hepatocytes to confirm the time- and dose-dependent anti-inflammatory effect of BBR. Consistent with the results from H4IIE cells, the phosphorylation state of p46 was decreased significantly in primary mouse hepatocytes upon BBR treatment for 1 hr, and, to a much larger extent, upon BBR treatment for 4 hr under both basal and palmitate-stimulated conditions (Fig. 5A). Unlike in H4IIE cells, BBR treatment markedly increased the phosphorylation state of AMPK in primary mouse hepatocytes. However, BBR stimulation of AMPK phosphorylation was not in a time-dependent manner, which was different from BBR suppression of p46 phosphorylation. In the dose-response study, treatment of primary mouse hepatocytes with BBR for 4 hr at a dose of 5 μM, 10 μM, or 25 μM effectively decreased the phosphorylation state of p46 in a dose-dependent manner under basal and/or palmitate-stimulated conditions. Upon treatment with BBR at various doses, AMPK phosphorylation was significantly increased, but not in a dose-dependent manner. When the mRNA levels of proinflammatory cytokines were measured, treatment with palmitate caused a significant increase in the mRNA levels of IL-1β, IL-6, and TNFα compared with control (BSA) (Fig. 5C), which was consistent with the proinflammatory effect of palmitate. Upon treatment with BBR, the mRNA levels of IL-6 and TNFα were nearly restored to the levels comparable with their respective levels in BSA-treated cells. Taken together, these results confirm that the anti-inflammatory effect of BBR appears to be independent of AMPK.

We also examined the direct effects of BBR on fat deposition in primary mouse hepatocytes. In the absence of BBR, treatment with palmitate caused a marked increase in fat deposition (Fig. 5D). This stimulatory effect of palmitate was nearly blunted by treatment with BBR. Consistent with changes in hepatocyte fat deposition, palmitate induced a marked increase in the mRNA levels of key lipogenic genes such as ACC, FAS, and SREBP1c compared with BSA (Fig. 5E). However, the effect of palmitate on inducing lipogenic gene expression was ameliorated upon treatment with BBR; although the mRNA levels of CPT1a were also decreased in response to BBR treatment.

### Berberine decreases obesity-associated adipose tissue inflammation without altering AMPK phosphorylation

**Figure 6:**
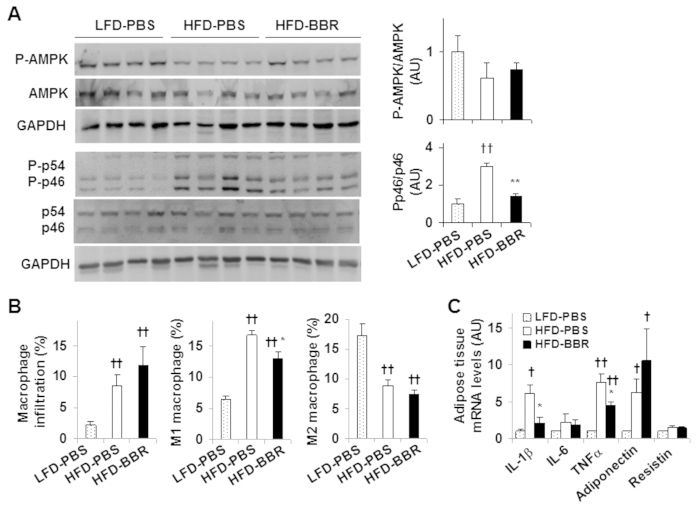
Berberine treatment decreases adipose tissue inflammatory responses without altering AMPK phosphorylation. Mice were treated as described in Fig. 1. After the feeding/treatment period, mice were fasted for 4 hr prior to collection of tissue samples. (**A**) Adipose tissue AMPK signaling and inflammatory signaling were examined using Western blot analyses. Ratios of phosphorylated AMPK to total AMPK (P-AMPK/AMPK) and phosphorylated JNK1 to total JNK1 (P-p46/p46) were quantified using densitometry and normalized to GAPDH (bar graphs). (**B**) Adipose tissue macrophage infiltration and polarization. Percentages of mature macrophages (F4/80^+^ CD11b^+^ cells) in adipose tissue stromal cells, as well as percentages of proinflammatory (M1, F4/80^+^ CD11b^+^ CD11c^+^ CD206^−^ cells) and anti-inflammatory (M2, F4/80^+^ CD11b^+^ CD11c^−^ CD206^+^ cells) among mature adipose tissue macrophages were calculated using FACS analyses. (**C**) The mRNA levels of adipose genes were quantified using real-time PCR. For bar graphs, data are means ± SE. n = 6–8). *P < 0.05 and **P < 0.01 HFD-BBR vs. HFD-PBS; ^†^*P* < 0.05 and ^††^*P* < 0.01 HFD-PBS or HFD-BBR vs. LFD-PBS.

Since obesity-associated dysfunctional adipose tissue contributes to a proinflammatory environment that exacerbates the progression of NAFLD^6^,^27^, we examined the effect of BBR on adipose tissue inflammation. In response to HFD feeding, the phosphorylation state of adipose tissue p46 (in HFD-PBS mice) was markedly increased compared with that in LFD-PBS mice, validating adipose tissue inflammation. Upon treatment with BBR, the phosphorylation state of adipose tissue p46 was decreased significantly (Fig. 6A). However, the phosphorylation state of adipose tissue AMPK in HFD-PBS mice only revealed an insignificant decrease compared with that in LFD-PBS mice. Also, treatment with BBR did not significantly increase the phosphorylation state of adipose tissue AMPK (Fig. 6A). Consistent with increased adipose tissue inflammatory signaling, percentages of mature macrophages and proinflammatory (M1) macrophages in adipose tissue of HFD-BBR mice were significantly higher than their respective percentages in LFD-PBS mice. However, in response to BBR treatment, percentages of M1 macrophages in adipose tissue of HFD-fed mice were significantly decreased in relative to those of HFD-PBS mice while macrophage infiltration and percentages of anti-inflammatory (M2) macrophages were not significantly altered (Fig. 6B). When proinflammatory cytokines were examined, the mRNA levels of IL-1β and TNFα in adipose tissue of HFD-PBS mice were significantly higher than their respective levels in LFD-PBS mice. Upon treatment with BBR, the mRNA levels of IL-1β and TNFα in adipose tissue of HFD-fed mice were significantly decreased in relative to their respective levels in HFD-PBS mice (Fig. 6C). Taken together, these results suggest that BBR decreases obesity-associated adipose tissue inflammation independent of AMPK phosphorylation.

## Discussion

Our previous^26^ and current studies showed that C57BL/6J mice fed an HFD developed obesity-associated insulin resistance, hepatic steatosis, and inflammation in both the liver and adipose tissue. Therefore, HFD-fed mice were used as a model of obesity-associated NAFLD to assess the beneficial effects of BBR. Consistent with several lines of studies^19^,^20^,^21^, BBR treatment not only improved HFD-induced hyperglycemia and glucose intolerance, but also significantly decreased the severity of liver and adipose tissue inflammation and hepatic steatosis. Using *in vitro* systems involving hepatoma cells and primary mouse hepatocytes, we confirmed that BBR had powerful anti-inflammatory effects on hepatocytes in both time-dependent and dose-dependent manners, and that BBR had a direct effect on blunting palmitate-induced hepatocyte fat deposition. Of significance, the effects of BBR on improving hepatic steatosis and on decreasing liver and adipose tissue inflammation in DIO mice were independent of AMPK. The same was found for the effect of BBR on suppressing inflammatory responses in hepatocytes. Therefore, the present study provides evidence to support that the anti-NAFLD actions of BBR were attributable to, in large extent, the anti-inflammatory effect of BBR that occurred in an AMPK-independent manner.

As a bioactive compound from a number of herbals that have long been used to treat inflammatory diseases, BBR is well documented to possess powerful anti-inflammatory properties. In support of this, BBR is capable of decreasing proinflammatory responses in a wide range of cells including macrophages, hepatocytes, and adipocytes^24^,^28^,^29^,^30^. Over the last two decades, the use of BBR has been extended to type 2 diabetes aiming at lowering hyperglycemia and hyperlipidemia^17^,^18^,^19^,^20^. More recently, BBR has also been used to treat fatty liver disease^19^,^20^. However, the extent to which the anti-inflammatory properties of BBR account for its anti-NAFLD actions remains to be explored. In the present study, BBR treatment significantly ameliorated HFD-induced inflammation in both the liver and adipose tissue as indicated by decreases in the proinflammatory signaling through p46 and in the mRNA levels of proinflammatory cytokines such as IL-1β, IL-6, and/or TNFα. Furthermore, treatment of two types of hepatocytes, hepatoma H4IIE cells and primary mouse hepatocytes, with BBR brought about similar effects on decreasing the proinflammatory signaling through p46 and/or the mRNA levels of IL-1β, IL-6, and TNFα. In addition, in both types of hepatocytes, the anti-inflammatory effect of BBR appeared to be both time-dependent and dose-dependent. Of importance, BBR treatment also brought about beneficial effects on hepatic steatosis in HFD-fed mice and on palmitate-induced fat deposition in hepatocytes. These effects were correlated well with the anti-inflammatory effects of BBR, but not AMPK phosphorylation state (see below). Considering the role of inflammation^31^,^32^,^33^,^34^,^35^,^36^, in particular the role for JNK1^31^,^34^, in promoting hepatic steatosis, the results from both *in vivo* and *in vitro* experiments of the present study indicate that BBR suppression of hepatocyte inflammatory responses critically contributes to a decrease in hepatocyte fat deposition, thereby hepatic steatosis.

While displaying anti-inflammatory effects, BBR treatment also increases liver AMPK phosphorylation (activation)^21^,^37^. When activated, AMPK is capable of suppressing lipogenesis through phosphorylating and inactivating the lipogenic enzyme ACC. In addition, a decrease in the production of malonyl-CoA due to AMPK inhibition of ACC brings about an increase in fatty acid oxidation via releasing the inhibitory effect of malonyl-CoA on CPT1a^38^. These combined effects of AMPK activation are considered to largely account for BBR actions on reducing hepatic steatosis as described by previous studies^21^,^23^. In the present study, however, AMPK appeared to not be involved in BBR actions as supported by two lines of evidence. First, treatment of DIO mice with BBR did not significantly alter the phosphorylation state of AMPK in both the liver and adipose tissue. Second, when the direct effects of BBR were examined, BBR did not significantly increase the phosphorylation state of AMPK at the time points where BBR decreased the phosphorylation state of p46 in a time-dependent manner. In addition, BBR did not increase the phosphorylation state of AMPK at the dose range where BBR decreased the phosphorylation state of p46 in a dose-dependent manner in H4IIE cells. Thus, BBR appeared to act through AMPK-independent mechanisms to decrease hepatic steatosis in DIO mice and to suppress palmitate-stimulated fat deposition in H4IIE cells. To be noted, metformin has been previously shown to suppress palmitate-stimulated fat deposition in H4IIE cells, and this effect of metformin was attributable to, at least in part, increased AMPK phosphorylation^26^. Upon carefully comparing the effects between BBR and metformin in H4IIE cells, we argue in favor that, unlike metformin actions, BBR suppression of hepatocyte fat deposition was associated with the anti-inflammatory effect of BBR, but not the AMPK phosphorylation state. This also explained well that the effects of BBR on decreasing HFD-induced hepatic steatosis were associated with a decrease in liver inflammation, but not AMPK phosphorylation.

Our findings on BBR regulation of AMPK phosphorylation were different from those reported by Brusq *et al*.^37^ and Kim *et al*.^21^. While further studies are needed to explore exactly why the discrepancies exist, it is possible that BBR regulation of AMPK phosphorylation varies depending on mouse models used and the methods of how BBR was delivered. In the study by Brusq *et al*. Syrian golden hamsters were fed an HFD for 2 weeks and used as a model of NAFLD whereas we used DIO mice. Much evidence has documented that hamsters are markedly different from mice. In the study by Kim *et al*. although obese mice were used as a model of NAFLD, BBR was delivered via intraperitoneal (IP) injections, but not oral gavages. Moreover, IP BBR injections brought about a decrease in body weight and an increase in whole body energy expenditure, which both may increase AMPK phosphorylation. In other words, the increase in liver AMPK phosphorylation observed by Kim *et al*. may be the secondary effects of BBR. Also, the study by Kim *et al*. did not validate the direct effect of BBR on altering AMPK phosphorylation in cultured hepatocytes. In contrast, we examined the direct effect of BBR in cultured hepatocytes, i.e., H4IIE cells and primary mouse hepatocytes, and our results suggest that the anti-inflammatory effects of BBR appear to be independent of AMPK phosphorylation. These findings were consistent with those observed in both HepG2 hepatocytes and C2C12 myotubes^39^, where BBR treatment promotes glucose consumption independent of AMPK. Given this, it is conceivable that activating AMPK is not a primary mechanism by which BBR exerts anti-NAFLD effects. However, our interpretation does not necessarily rule out a contribution of AMPK activation to, at a certain extent, the beneficial effects of BBR.

The role for obesity-associated dysfunctional adipose tissue in the development of NAFLD also has been well established. On the one hand, adipose tissue delivers excessive free fatty acids to the liver, thereby facilitating hepatic steatosis. On the other hand, adipose tissue secretes proinflammatory mediators that trigger or exacerbate liver inflammation as a key element of NAFLD. In the present study, treatment of DIO mice with BBR did not alter visceral fat mass and adiposity. In contrast, BBR treatment ameliorated adipose tissue inflammatory responses as indicated by significant decreases in the proinflammatory signaling through p46, percentages of M1 macrophages, and the mRNA levels of proinflammatory cytokines. Clearly, these results suggest an involvement of improved adipose tissue inflammation in the beneficial effects of BBR on aspects of NAFLD. However, the proportional contribution of improved inflammation in the liver and adipose tissue to the overall improvement of NAFLD in DIO mice is not known, and may warrant future investigations.

In summary, the present study provided evidence to support the potential therapeutic effects of BBR on reducing liver inflammation and steatosis while improving systemic insulin sensitivity and glucose homeostasis. The mechanisms underlying the beneficial effects of BBR could result largely from the anti-inflammatory effect of BBR. In addition, BBR appears to have a limited role in increasing AMPK phosphorylation state in the context of NAFLD. Taken together, our results validate the potential of using BBR for treatment and/or prevention of obesity-associated NAFLD.

## Materials and Methods

### Animal experiments

C57BL/6J mice were obtained from the Jackson Laboratory and maintained on a 12:12-h light-dark cycle (lights on at 06:00). At 5–6 weeks of age, male mice were fed a high-fat diet (HFD, 60% fat calories, 20% protein calories, and 20% carbohydrate calories) for 12 weeks and treated with BBR (100 mg/kg body weight/d, suspension in PBS) or PBS via oral gavages for the last 4 weeks. The dose of BBR was chosen based on our pilot study and published literature^37^. As additional controls, gender- and age-matched C57BL/6J mice were fed a low-fat diet (LFD, 10% fat calories, 20% protein calories, and 70% carbohydrate calories) for 12 weeks and treated with PBS for the last 4 weeks. Both diets are products of Research Diets, Inc (New Brunswick, NJ) and contain the same amount of casein, L-cysteine, cellulose, sucrose, soybean oil, and minerals. However, the HFD contains much more lard but none corn starch compared with the LFD. During the 12-week feeding/treatment period, body weight and food intake of the mice were recorded weekly. After the feeding/treatment period, mice were fasted for 4 hr before sacrifice for collection of blood and tissue samples^25^,^40^,^41^. Liver weight was recorded. Also, epididymal, mesenteric, and perinephric fat depots were dissected and weighed as visceral fat content^25^. After weighing, part of epididymal fat was subjected to isolation of stromal vascular cells as described below. Additional liver and adipose tissue samples were either fixed and embedded for histological analyses (H&E staining) or frozen in liquid nitrogen and stored at −80 °C for further analyses^25^. Some mice were fasted similarly and used for insulin and glucose tolerance tests as described below. All animals received human care and all study protocols were approved by the Institutional Animal Care and Use Committee of Texas A&M University. In addition, all experiments were performed in accordance with relevant guidelines and regulations.

### Insulin and glucose tolerance tests

Mice were fasted for 4 hr and received an intraperitoneal injection of insulin (1 U/kg body weight) or D-glucose (2 g/kg body weight). For insulin tolerance tests, blood samples (5 μl) were collected from the tail vein before and at 15, 30, 45, and 60 min after the bolus insulin injection. Similarly, for glucose tolerance tests, blood samples were collected from the tail vein before and at 30, 60, 90 and 120 min after the glucose bolus injection^42^,^43^. The levels of plasma glucose were measured using an enzymatic assay kit (Sigma, St. Louis, MO).

### Isolation of stromal vascular cells from adipose tissue

Adipose tissue stromal vascular cells (SVC) were isolated using the collagenase digestion method as previously described^42^,^44^. After digestion and centrifugation, the pelleted cells were collected as SVC and subjected to FACS analyses.

### Flow cytometry analysis

Adipose tissue SVC were stained with fluorescence-tagged antibodies: anti-F4/80, anti-CD11b for macrophages, and anti-CD11c and anti-CD206 for macrophage inflammatory status as previously described^45^, and subjected to FACS analyses using BD Accuri C6 flow cytometer (BD Biosciences, San Jose, California, USA).

### H&E and Oil-Red-O staining

The paraffin-embedded livers were cut into sections of 5 μm thickness and stained with hematoxylin and eosin (H&E) while frozen liver sections were stained with Oil-Red-O as previously described^46^.

### Cell culture and treatment

H4IIE cells (rat hepatoma cells) were maintained in high glucose Dulbecco’s modified eagle medium (DMEM) supplemented with 10% fetal bovine serum, 100 units/ml penicillin and 100 μg/l streptomycin as previously described^15^,^16^. At 80% confluence, H4IIE cells were subjected to the following studies. In a time-course study, H4IIE cells were pre-treated with palmitate (250 μM, conjugated with BSA) or BSA for 2 hr and then supplemented with BBR (25 μM in PBS) or PBS for an additional 10 min, 1 hr, 4 hr, 8 hr, and/or 24 hr. For a dose-response study, H4IIE cells were pre-treated with palmitate (250 μM) or BSA for 2 hr and then supplemented with BBR or PBS for an additional 24 hr. In addition, primary mouse hepatocytes were isolated as previously described^15^,^16^ and subjected to time-course and dose-response studies in the same ways for H4IIE cells. Briefly, after attachment, primary mouse hepatocytes were pre-treated with palmitate (250 μM) for 2 hr and then supplemented with BBR (25 μM) or PBS for an additional 1 hr and/or 4 hr. In a dose-response study, primary mouse hepatocytes were pre-treated with palmitate (250 μM) or BSA for 2 hr and then supplemented with BBR at a dose of 5, 10, or 25 μM or PBS for an additional 4 hr. Additional primary hepatocytes were treated with or without palmitate for 2 hr in the presence or absence of BBR for an additional 4 hr to measure the mRNA levels of pro-inflammatory cytokines and metabolic genes. To examine lipid accumulation, the treated hepatocytes were stained with Oil-Red-O for the last 1 hr.

### Western blots

Lysates were prepared from frozen tissue samples and cultured cells using the lysis buffer containing 50 mm HEPES (pH 7.4), 1% Triton X-100, 50 mm sodium pyrophosphate, 0.1 m sodium fluoride, 10 mm EDTA, 10 mm sodium orthovanadate, 10 μg/ml aprotinin, 10 μg/ml leupeptin, 2 mm benzamidine, and 2 mm phenylmethylsulfonyl fluoride. After protein electrophoresis and transfer, immunoblots were performed using rabbit anti-serum as primary antibody at a 1:1,000 dilution. The blot was followed by a 1:10,000 dilution of goat anti-rabbit horseradish peroxidase-conjugated secondary antibody kit (Immobilon™ Western; EMD Millipore, Billerica, MA, USA) as previously described^25^. GAPDH was used as a loading control. The maximum intensity of each band was quantified using ImageJ software. Ratios of P-AMPK/AMPK, P-ACC/ACC, and P-p46/p46 were normalized to GAPDH and adjusted relative to the average of PBS-treated control, which was arbitrarily set as 1 (AU).

### RNA isolation, reverse transcription, and real-time PCR

The total RNA was isolated from frozen tissue samples and cultured/isolated cells. Reverse transcription was performed using the GoScript™ Reverse Transcription System (Promega) and real-time PCR analysis was performed using SYBR Green (LightCycler® 480 system; Roche)^16^,^41^,^47^. The mRNA levels were analyzed for ACC1, FAS, CPT1a, SREBP1c, IL-1β, IL-6, TNFα, adiponectin, and/or resistin in tissue and/or cell samples. A total of 0.1 μg RNA was used for the determination. Results were normalized to 18s ribosomal RNA as plotted as relative expression to the average of PBS-treated control, which was set as 1.

### Statistical Methods

Numeric data are presented as means ± SE (standard error). Two-tailed Student’s *t* tests and/or two-tailed ANOVA were used for statistical analyses. Differences were considered significant at the *P* < 0.05.

## Additional Information

**How to cite this article**: Guo, T. *et al*. Berberine Ameliorates Hepatic Steatosis and Suppresses Liver and Adipose Tissue Inflammation in Mice with Diet-induced Obesity. *Sci. Rep.* **6**, 22612; doi: 10.1038/srep22612 (2016).
